# Feasibility of Peroral Cholangioscopy in the Initial Endoscopic Retrograde Cholangiopancreatography for Malignant Biliary Strictures

**DOI:** 10.3390/diagnostics14222589

**Published:** 2024-11-18

**Authors:** Yuichi Suzuki, Tomohiro Ishii, Haruo Miwa, Takeshi Sato, Yoshihiro Goda, Kuniyasu Irie, Kazuya Sugimori, Shin Maeda

**Affiliations:** 1 Department of Gastroenterology, Yokohama City University Graduate School of Medicine, Yokohama 236-0004, Japan; suzuki.yui.ar@yokohama-cu.ac.jp (Y.S.); tak_sato@yokohama-cu.ac.jp (T.S.); y_gouda@yokohama-cu.ac.jp (Y.G.); k_irie@yokohama-cu.ac.jp (K.I.); smaeda@yokohama-cu.ac.jp (S.M.); 2 Gastroenterological Center, Yokohama City University Medical Center, Yokohama 232-0024, Japan; 3 Department of Gastroenterology, Saiseikai Yokohamashi Nanbu Hospital, Yokohama 234-0054, Japan; ishiito@nanbu.saiseikai.or.jp (T.I.); sugimori@yokohama-cu.ac.jp (K.S.)

**Keywords:** cholangioscopy, endoscopic retrograde cholangiopancreatography, malignant biliary stricturey, feasibility

## Abstract

**Background:** Peroral cholangioscopy (POCS) is valuable for assessing malignant biliary strictures; however, biliary drainage prior to POCS often hinders accurate diagnosis. **Objectives:** This retrospective study aimed to investigate the feasibility of POCS using a newly developed cholangioscope, CHF-B290, during initial endoscopic retrograde cholangiopancreatography (ERCP) for malignant biliary strictures. **Methods:** This multicenter retrospective study included patients who underwent initial ERCP for malignant biliary strictures at two institutions between January 2018 and March 2022. Patients who underwent initial ERCP with POCS were classified into the POCS group, and those without POCS were classified into the non-POCS group. To prevent post-POCS cholangitis, the original irrigation system for CHF-B290 was used in all POCS examinations. The primary endpoint was the rate of post-ERCP biliary infections, and the secondary endpoints were other ERCP-related complications, including pancreatitis, bleeding, and perforation. **Results:** Overall, 53 and 94 patients were included in the POCS and non-POCS groups, respectively. For the primary endpoint, the rate of post-ERCP biliary infection was not significantly different between the two groups (1.9% vs. 5.3%, *p* = 0.42). For the secondary endpoints, no significant differences were observed in the rates of post-ERCP pancreatitis (5.7% vs. 6.4%, *p* = 1.00) and other ERCP-related complications. The overall complication rate was 9.4% in the POCS group and 13% in the non-POCS group (*p* = 0.60). **Conclusions:** POCS during the initial ERCP for malignant biliary strictures is feasible.

## 1. Introduction

Peroral cholangioscopy (POCS) allows for direct visualization of the bile duct and targeted biopsies. The primary indications for POCS are evaluation of indeterminate biliary strictures, assessment of the extent of cholangiocarcinoma, and treatment of choledocholithiasis. Recently, a digital single-operator cholangioscope (SpyGlass DS; Boston Scientific Corporation, Marlborough, Massachusetts, United States) has been reported to be a valuable diagnostic tool for evaluating malignant biliary stricture [1,2,3,4]. The SpyGlass DS system has the advantage of maneuverability inside the bile duct; however, its image quality and cost-effectiveness are inferior to those of a conventional mother–baby cholangioscope (CHF-B260; Olympus Corporation, Tokyo, Japan) [5,6]. In 2019, a newly developed mother–baby digital cholangioscope (CHF-B290; Olympus Corporation, Tokyo, Japan) became available. This cholangioscope has high imaging quality and improved durability compared to the CHF-B260 cholangioscope. Moreover, new image enhancement systems are available with the EVIS X1 system (Olympus Corporation, Tokyo, Japan) [7,8].

**Figure 1:**
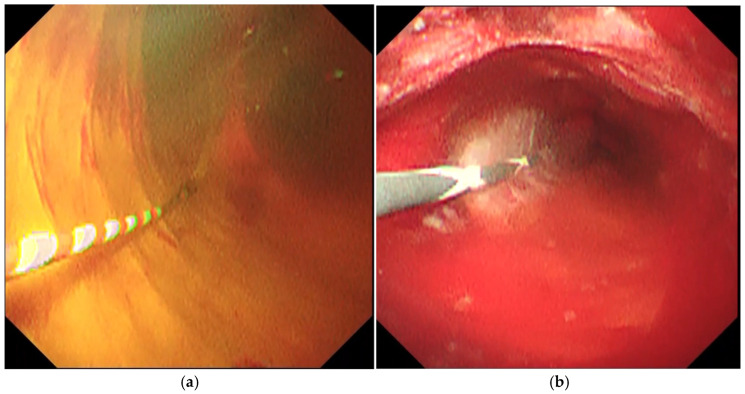
Peroral cholangioscopy (POCS) images before (**a**) and after (**b**) biliary stenting in the same case.

**Figure 2:**
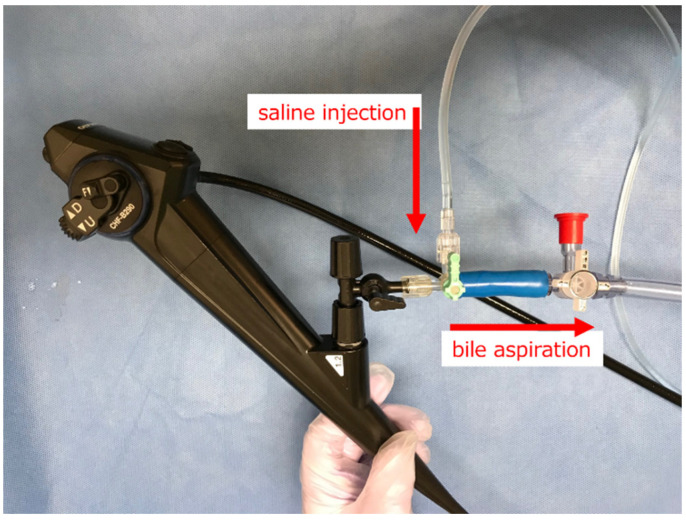
Original irrigation system for CHF-B290.

When using POCS, it is necessary to irrigate the bile duct with saline solution to remove sludge, mucus, and the contrast agent. However, excessive intrabiliary pressure can lead to POCS-related cholangitis [9,10,11]. In particular, the mother–baby cholangioscope has a lower irrigation ability than the SpyGlass DS owing to a single working channel. To prevent POCS-related cholangitis, endoscopic drainage is often performed prior to endoscopic retrograde cholangiopancreatography (ERCP) with POCS; however, biliary stenting for malignant biliary strictures often causes inflammatory reactions or bleeding in the bile duct wall [12,13], hindering an accurate diagnosis of POCS [14] (Figure 1). To overcome this disadvantage, we developed an original irrigation system for CHF-B290 and performed a safe and efficient POCS examination during the initial ERCP for malignant biliary strictures (Figure 2). The aim of this study was to investigate the feasibility of using POCS with CHF-B290 during the initial ERCP for malignant biliary strictures.

## 2. Materials and Methods

### 2.1. Study Design and Patients

This multicenter retrospective study included patients who underwent initial ERCP for malignant biliary strictures between January 2018 and March 2022. All ERCPs were performed at Saiseikai Yokohamashi Nanbu Hospital and Yokohama City University Hospital. Patients who underwent an initial ERCP with POCS were classified into the POCS group, and those who underwent an initial ERCP without POCS were classified into the non-POCS group.

Patients with severe cholangitis according to the Tokyo Guidelines 2018 (TG18) [15]; pancreatitis before ERCP; or surgically altered anatomy, except for Billroth I reconstruction, were excluded. Patient characteristics, laboratory data, imaging findings, endoscopic procedures, histopathology, adverse events, and clinical course after ERCP were analyzed using medical records.

### 2.2. Original Irrigation System

Because CHF-B290 lacks a dedicated irrigation channel, we developed an original irrigation system to perform a safe and efficient POCS examination (Figure 2). A three-way valve connected the saline injection and aspiration tubes for irrigation. A large-diameter tube (30Fr) facilitated efficient bile aspiration. An irrigation system was utilized for all POCS examinations.

### 2.3. Procedures

All procedures were performed using a duodenoscope (TJF-Q290V, TJF-260V, and JF-260V; Olympus Corporation, Tokyo, Japan), and all POCS examinations were performed using CHF-B290. In both groups, all procedures were supervised by an expert with over 10 years of experience in ERCP. Prophylactic antibiotics, including Cefmetazole and Sulbactam/Cefoperazone, were routinely administered before the ERCP.

During ERCP, biliary cannulation and cholangiography were performed initially, followed by intraductal ultrasonography as needed. Subsequently, endoscopic sphincterotomy (EST) was performed prior to biopsy or POCS examination, unless the patient received antithrombotic therapy. In the non-POCS group, fluoroscopy-guided biopsy with Radial Jaw biopsy forceps (Boston Scientific) was performed after EST. In the POCS group, CHF-B290 was inserted through the bile duct strictures after EST. Bile aspiration was conducted for several minutes using the original irrigation system until visual observation indicated that the bile duct lumen collapsed or no further bile could be aspirated. Subsequently, saline was injected. After obtaining a clear optical field, POCS was performed to evaluate the presence or extent of malignancy of the biliary mucosa. Fluoroscopy-guided biopsy for biliary strictures was performed to obtain large specimens after POCS observation. Biliary stenting was performed as required in both groups.

### 2.4. Definitions and Outcomes

Difficult biliary cannulation is defined as the inability to achieve selective biliary cannulation by standard ERCP techniques within 10 min [16]. The severity of pre-ERCP cholangitis was defined and graded using the TG18 scale as mild, moderate, or severe. Patients with elevated C-reactive protein (CRP) and hepatobiliary enzyme levels, even without fever, were diagnosed with pre-ERCP cholangitis. The complications and severity of cholangitis, pancreatitis, bleeding, and perforation related to ERCP were defined and graded according to the Cotton classification [17]. Post-ERCP cholangitis was diagnosed by developing a fever exceeding 38 °C for 24–48 h.

The primary endpoint of this study was the rate of post-ERCP biliary infections in the POCS group compared with that in the non-POCS group. The secondary endpoint was the incidence of other ERCP-related complications, including pancreatitis, bleeding, and perforation. The complication rates in a subset of patients with pre-ERCP cholangitis were also evaluated.

### 2.5. Statistical Analysis

We performed Fisher’s exact test for nominal variables and *t*-test for continuous variables. Statistical significance was set at *p* < 0.05. All statistical analyses were performed using EZR software version 4.0.3 (Saitama Medical Center, Jichi. Medical University, Saitama, Japan) [18].

### 2.6. Ethics

The study protocol was approved by the Institutional Review Boards of Yokohama City University Hospital and Saiseikai Yokohamashi Nanbu Hospital. The requirement for patient consent was waived because of the retrospective nature of the study. Information is shared through an opt-out process. All authors had full access to all data in the study and had the final responsibility for the decision to submit the manuscript for publication.

## 3. Results

### 3.1. Patients

Overall, 53 and 94 patients were included in the POCS and non-POCS groups, respectively. The patient characteristics are shown in Table 1. The median ages of the POCS and non-POCS groups were 78 (37–95) and 77 (51–97) years, respectively. The baseline characteristics were not significantly different between the two groups in terms of age, sex, age-adjusted Charlson comorbidity index (CCI), Eastern Cooperative Oncology Group performance status (ECOG PS), or rate of antithrombotic therapy. The number of patients with pre-ERCP cholangitis was 27 (51%) in the POCS group and 42 (45%) in the non-POCS group (*p* = 0.50), including patients with moderate cholangitis (20 [38%] in the POCS group and 25 [27%] in the non-POCS group (*p* = 0.19). Blood cultures were performed primarily in patients with a fever or elevated inflammatory markers prior to ERCP, according to standard clinical practice. Consequently, blood cultures were collected in 20 patients in the POCS group and 21 patients in the non-POCS group. Among these, positive blood cultures were observed in three patients (5.7%) in the POCS group and four patients (4.3%) in the non-POCS group (*p* = 0.70). Table 1 also shows the primary diseases, including biliary tract cancer, pancreatic cancer, hepatocellular cancer, and other malignant diseases. No significant differences in primary diseases were identified between the two groups.

Details of the laboratory data before ERCP are presented in Table 2. There were no significant differences in laboratory data between the two groups. As all patients were examined prior to undergoing biliary drainage, most had jaundice. The median total bilirubin value was 7.0 mg/dL in the POCS group and 5.5 mg/dL in the non-POCS group (*p* = 0.44).

### 3.2. Procedures and Outcomes

The details of the procedure are presented in Table 3. The median cannulation time was not significantly different between the two groups (17 min [1–48 min] vs. 10 min [1–83 min], *p* = 0.81). In addition, the rate of patients with difficult biliary cannulation was not significantly different between the two groups (64% vs. 52%, *p* = 0.17). The median procedure time for POCS was 10 (1–37) min. The median ERCP procedure time in the POCS group (67 min [37–124 min]) was significantly longer than that in the non-POCS group (50 min [14–110 min]) (*p* < 0.01). EST was performed more frequently in the POCS group (*p* < 0.01), whereas pancreatic stenting was performed more frequently in the non-POCS group (*p* < 0.01). The rate of successful biliary stent deployment at the intended position was 96% in the POCS group and 93% in the non-POCS group, with no significant difference observed. There were no significant differences in other endoscopic procedures between the two groups.

### 3.3. Complications

The complications associated with ERCP are shown in Table 4. For the primary endpoint, the incidence of post-ERCP biliary infections was not significantly different between the two groups (1.9% vs. 5.3%, *p* = 0.42). In the non-POCS group, acute cholecystitis caused by metallic biliary stenting over the orifice of the cystic duct was observed in two patients who required percutaneous transhepatic gallbladder drainage after ERCP. Regarding the secondary endpoints, the incidence of other ERCP-related complications was not significantly different between the two groups, including post-ERCP pancreatitis (5.7% vs. 6.4%, *p* = 1.00). The overall complication rate was 9.4% (5/53) in the POCS group and 13% (12/94) in the non-POCS group (*p* = 0.60).

Regarding the difficult biliary cannulation cases, all five cases with complications were in the difficult cannulation category in the POCS group. In the non-POCS group, six cases (two with biliary infection, three with pancreatitis, and one with perforation) were classified as difficult cannulation cases.

In the subset of patients with pre-ERCP cholangitis, no post-ERCP biliary infections were observed in the POCS group (27 patients), whereas post-ERCP cholangitis was observed in 3 of 42 patients (7.1%) in the non-POCS group (*p* = 0.28).

## 4. Discussion

POCS has been widely used for the investigation of biliary diseases over the past decade, especially after digital single-operator POCS became available. Several studies have shown that ERCP with POCS is effective for the assessment of indeterminate biliary strictures, targeted biopsy, and treatment of difficult bile duct stones with electrohydraulic or laser lithotripsy [1,3,19,20,21]. However, the feasibility of performing POCS during the initial ERCP for malignant biliary obstruction remains unclear. In this study, POCS in the initial ERCP for malignant biliary strictures did not increase the risk of post-ERCP biliary infections or other complications compared with ERCP without POCS. The results of our study show that POCS during the initial ERCP for malignant biliary strictures is feasible.

Most previous studies have reported the usefulness and feasibility of the digital single-operator cholangioscope [1,2,3,4]. One of the features of our study was the use of the newly developed mother–baby digital cholangioscope, CHF-B290. CHF-B290 has a higher imaging quality than SpyGlass DS, and narrow-band imaging (NBI) can be used. When used with the EVIS X1 system, it supports advanced imaging options such as red dichromatic imaging (RDI), and texture and tone enhancement imaging (TXI), which aid in the detailed visualization of biliary structures. Furthermore, CHF-B290 is more cost-effective than SpyGlass DS, making it an appealing option for broader clinical use. However, CHF-B290 has a lower irrigation capability owing to its single working channel. To compensate for this disadvantage, an original irrigation system was developed for CHF-B290 and used in all POCS examinations. In this study, the overall complication rate in the POCS group was 9.4%, which was within the acceptable range compared to previously reported rates of 5–10% [11,19,22,23,24,25,26]. Regarding the primary endpoint, post-ERCP biliary infection was the most concerning complication resulting from POCS examination [27,28]; however, the incidence rate in the POCS group (1.9%) was not significantly different from that in the non-POCS group (5.3%). This result is comparable to those of previous reports [19,22,23]; however, these reports did not necessarily include only patients who underwent POCS during the initial ERCP. Furthermore, in our study, post-ERCP cholangitis did not increase in the POCS group, even in patients with pre-ERCP cholangitis of up to the moderate grade. In our study, a relatively large number of patients were classified as having pre-ERCP cholangitis. According to TG18, patients with simultaneous elevation of CRP and hepatobiliary enzymes are diagnosed with acute cholangitis. Additionally, the severity of cholangitis is classified as moderate in older adult patients due to the presence of hyperbilirubinemia or hypoalbuminemia, even without a high fever. Therefore, the population with pre-ERCP cholangitis may have been overestimated due to tumor-related inflammation. The low incidence of post-ERCP cholangitis in the POCS group was attributed to the original irrigation system, which facilitated efficient bile aspiration and saline irrigation. Excessive pressure in the bile duct during ERCP causes post-ERCP cholangitis and sepsis [9,10,11]. To prevent this complication, bile aspiration and avoidance of excessive contrast injections are important [29,30]. Although CHF-B290 has a lower irrigation ability than SpyGlass DS, the original irrigation system, which allows for efficient bile aspiration and irrigation, successfully prevents post-ERCP cholangitis. Furthermore, prophylactic antibiotics before POCS may have contributed to the low incidence of cholangitis in our study, as reported in a previous prospective study [10]. The study revealed that true-positive blood cultures were noted in 20 (27.8%) of the 72 patients who underwent ERCP with POCS, and 10 patients (13.9%) had sustained bacteremia related to POCS. In our study, prophylactic antibiotics were routinely administered to all patients before ERCP. Regarding the secondary endpoints, post-ERCP pancreatitis was another concerning complication of POCS; however, the incidence rate in the POCS group (5.7%) was not significantly different from that in the non-POCS group (6.4%). This result is slightly higher than those of previous reports [11,22,23]; however, the patient characteristics in our study were different from these reports. To prevent pancreatitis, EST was performed sufficiently before POCS in our cohort. In addition, since our study was conducted on cases with naïve papilla, the difficulty of selective biliary cannulation may have influenced the incidence of complications. A recent network meta-analysis demonstrated that early needle-knife techniques and transpancreatic sphincterotomy are superior to other interventions in decreasing post-ERCP pancreatitis rates for patients with difficult cannulation [31]. Although there were no significant differences in the rate of patients who underwent precutting between the two groups in our cohort, the rate of post-ERCP pancreatitis may have been influenced by the rate of precutting.

Another feature of our study was that it included various types of malignant diseases. First, unlike most previous studies that included both benign and malignant biliary strictures [4,32], our study focused only on patients with malignant biliary strictures. One of the main indications for POCS is the evaluation of cholangiocarcinoma. Assessing distal biliary strictures with POCS is particularly challenging due to difficulties in maintaining stable scope positioning, which often limits accurate imaging and tissue acquisition. Previous studies have indicated that the tissue adequacy of POCS-guided biopsies from distal biliary strictures is lower than that from other locations [24,33], constraining the evaluation of distal lesions. However, for surgical planning, accurately diagnosing the lateral extent of proximal spread in cholangiocarcinoma is crucial. Therefore, our study focused on evaluating the proximal bile duct in patients with distal biliary strictures to enhance diagnostic reliability for surgical decisions. Second, our study included a relatively large number of patients with malignant diseases other than cholangiocarcinoma. Although the evaluation of cholangiocarcinoma is a primary indication for POCS, the etiology of malignant biliary strictures is often unknown at the initial ERCP. To ensure an accurate diagnosis, POCS should be performed before biliary stenting. CHF-B290 is easy to use in the initial ERCP for various types of biliary strictures because it is reusable and cost-effective. Therefore, we performed POCS in patients other than cholangiocarcinoma. As a result, we were able to evaluate the extent of the lesion, exposure of the tumor to the mucosal surface, and presence of tumor invasion into the cystic duct. When tumor exposure in the bile duct was not observed, endoscopic ultrasonography-guided tissue acquisition was performed. POCS is valuable for detecting subtle changes in the biliary mucosa, both for assessing the superficial spread of biliary tract cancer and for identifying the infiltration of other malignant diseases. Therefore, clarifying the feasibility of POCS during initial ERCP is valuable.

Our study had some limitations. First, this was a retrospective study with a small sample size. Further prospective studies are required to confirm these results. In addition, the study period differed between the two groups. Therefore, the results of our study have a possible risk of type II errors and selection bias, even though consecutive ERCP cases were enrolled in both groups. Second, only patients with malignant biliary strictures were included in this study. Therefore, it differs from clinical practice, where cases of benign biliary strictures are also present. Third, this study did not compare the feasibility of CHF-B290 with the older cholangioscope CHF-B260. However, the feasibility of CHF-B260 has been verified in previous reports, and CHF-B290 is the upgraded model of CHF-B260. There are several changes from CHF-B260 to CHF-B290 that are considered to reduce the risk of post -ERCP cholangitis. In addition, CHF-B290 has been improved in image quality and durability, and new image enhancement systems are available with the EVIS X1 system. From these backgrounds, our study is valuable in demonstrating the feasibility of the new mother–baby cholangioscope even without a comparison with CHF-B260. Finally, procedural details, such as the number of patients who underwent EST or pancreatic stenting, were significantly different between the two groups, which may have influenced the ERCP-associated complication rates.

## 5. Conclusions

To the best of our knowledge, this is the first study to investigate the feasibility of POCS in the initial ERCP for malignant biliary strictures. Although the indications for POCS should be carefully considered, our study suggests that POCS during the initial ERCP for malignant biliary strictures is feasible. Unless patients have severe cholangitis, POCS examination should be considered in the initial ERCP for an accurate diagnosis.

## Figures and Tables

**Table 1:** Patients’ characteristics.

	POCS (*n* = 53)		Non-POCS (*n* = 94)		*p*-Value
Age, years, median (range)	78	(37–95)	77	(51–97)	0.86
Sex, female, *n* (%)	19	(36)	44	(47)	0.23
Age-adjusted CCI, median (range)	5	(1–8)	5	(2–9)	0.62
ECOG PS, *n* (%)					0.22
0	42	(79)	67	(71)	
1	9	(17)	16	(17)	
2	2	(3.8)	9	(9.6)	
3	0	(0)	2	(2.1)	
4	0	(0)	0	(0)	
Antithrombotic drug, *n* (%)	15	(28)	16	(17)	0.14
Pre-ERCP cholangitis, *n* (%)	27	(51%)	42	(45%)	0.50
Mild	7	(13%)	17	(18%)	0.50
Moderate	20	(38%)	25	(27%)	0.19
Positive blood cultures, *n* (%)	3	(5.7%)	4	(4.3%)	0.70
Disease, *n* (%)					
Pancreatic caner	28	(53)	42	(45)	0.39
Biliary tract cancer	21	(40)	43	(46)	0.49
Extrahepatic bile duct cancer	18	(34)	30	(32)	0.86
Distal	10		21		
Perihilar	8		9		
Gallbladder cancer	1	(1.9)	6	(6.4)	0.42
Ampullary cancer	0	(0)	6	(6.4)	0.09
Intrahepatic cholangiocarcinoma	2	(3.8)	1	(1.1)	0.30
Hepatocellular cancer	1	(1.9)	0	(0)	0.36
Other malignant disease	3	(5.7)	9	(9.6)	0.54

POCS: peroral cholangioscopy, CCI: Charlson comorbidity index, ECOG PS: Eastern Cooperative Oncology Group performance status.

**Table 2:** Pre-ERCP laboratory data.

	POCS (*n* = 53)		Non-POCS (*n* = 94)		*p*-Value
WBC, /μL, median (range)	6000	(3100–30,900)	5400	(2700–19,600)	0.21
Hb, g/dL, median (range)	12.3	(6.7–16.8)	12.0	(7.9–15.1)	0.37
Plt, ×10^4^/μL, median (range)	22.4	(11.7–49.3)	24.3	(5.0–43.4)	0.85
Cre, mg/dL, median (range)	0.82	(0.42–1.91)	0.74	(0.41–3.72)	0.74
Alb, g/dL, median (range)	3.1	(1.6–4.5)	3.2	(1.8–4.4)	0.09
T-Bil, mg/dL, median (range)	7.0	(0.4–31.2)	5.5	(0.4–24.6)	0.44
AST, U/L, median (range)	110	(18–877)	137	(16–547)	0.26
ALT, U/L, median (range)	137	(11–1188)	168	(12–897)	0.42
CRP, mg/dL, median (range)	1.27	(0.03–20.2)	1.04	(0.06–41.7)	0.94

WBC: white blood cell count, Hb: hemoglobin, Plt: platelet, Cre: creatinine, Alb: albumin, T-Bil: total bilirubin, AST: aspartate aminotransferase, ALT: alanine aminotransferase, CRP: C-reactive protein.

**Table 3:** Details in procedure.

	POCS (*n* = 53)		Non-POCS (*n* = 94)		*p*-Value
Cannulation time, min, median (range)	17	(1–48)	10	(1–83)	0.81
Difficult biliary cannulation, *n* (%)	34	(64)	49	(52)	0.17
Procedure time of POCS, min, median (range)	10	(1–37)	-	-	-
Procedure time of ERCP, min, median (range)	67	(37–124)	50	(14–110)	<0.01
Endoscopic sphincterotomy, *n* (%)	52	(98)	65	(69)	<0.01
Precutting, *n* (%)	2	(3.8)	11	(12)	0.14
Endoscopic papillary balloon dilation, *n* (%)	0	(0)	2	(2.1)	0.54
Pancreatic duct injection, *n* (%)	9	(17)	27	(29)	0.16
Biliary stent, *n* (%)	51	(96)	87	(93)	0.49
Plastic stent	51	(96)	83	(88)	
Metallic stent	0	(0)	4	(4.3)	
Pancreatic stent, *n* (%)	1	(1.9)	17	(18)	<0.01

POCS: peroral cholangioscopy, ERCP: endoscopic retrograde cholangiopancreatography.

**Table 4:** Complications.

	POCS (*n* = 53)		Non-POCS (*n* = 94)		*p*-Value
Total, *n* (%)	5	(9.4)	12	(13)	0.60
Biliary infection, *n* (%)	1	(1.9)	5	(5.3)	0.42
Cholangitis	1		3		
Cholecystitis	0		2		
Mild	1		1		
Moderate	0		4		
Severe	0		0		
Pancreatitis, *n* (%)	3	(5.7)	6	(6.4)	1.00
Mild	2		3		
Moderate	1		3		
Severe	0		0		
Bleeding, *n* (%)	1	(1.9)	0	(0)	0.36
Mild	0				
Moderate	1				
Severe	0				
Perforation, *n* (%)	0	(0)	1	(1.1)	1.00
Mild			0		
Moderate			0		
Severe			1		

## Data Availability

The datasets generated during and/or analyzed during this study are available from the corresponding author upon reasonable request.

## References

[1] Gerges C., Beyna T., Tang R.S.Y., Bahin F., Lau J.Y.W., van Geenen E., Neuhaus H., Nageshwar Reddy D., Ramchandani M. (2020). Digital single-operator peroral cholangioscopy-guided biopsy sampling versus ERCP-guided brushing for indeterminate biliary strictures: A prospective, randomized, multicenter trial (with video). Gastrointest. Endosc..

[2] Shah R.J., Raijman I., Brauer B., Gumustop B., Pleskow D.K. (2017). Performance of a fully disposable, digital, single-operator cholangiopancreatoscope. Endoscopy.

[3] Turowski F., Hugle U., Dormann A., Bechtler M., Jakobs R., Gottschalk U., Notzel E., Hartmann D., Lorenz A., Kolligs F. (2018). Diagnostic and therapeutic single-operator cholangiopancreatoscopy with SpyGlassDS: Results of a multicenter retrospective cohort study. Surg. Endosc..

[4] Ogura T., Imanishi M., Kurisu Y., Onda S., Sano T., Takagi W., Okuda A., Miyano A., Amano M., Nishioka N. (2017). Prospective evaluation of digital single-operator cholangioscope for diagnostic and therapeutic procedures (with videos). Dig. Endosc..

[5] Kanno Y., Koshita S., Ogawa T., Masu K., Kusunose H., Sakai T., Murabayashi T., Haegawa S., Kozakai F., Yonamine K. (2018). Peroral cholangioscopy by SpyGlass DS versus CHF-B260 for evaluation of the lateral spread of extrahepatic cholangiocarcinoma. Endosc. Int. Open.

[6] Ishida Y., Itoi T., Okabe Y. (2016). Types of Peroral Cholangioscopy: How to Choose the Most Suitable Type of Cholangioscopy. Curr. Treat. Options Gastroenterol..

[7] Ishii T., Kaneko T., Murakami A., Ueda M., Sugimori K., Kawana I., Maeda S. (2023). New image-enhanced cholangioscopy for the diagnosis of cholangiocarcinoma. Endoscopy.

[8] Ishii T., Kaneko T., Murakami A., Enomoto M., Sugimori K., Kawana I., Maeda S. (2023). Cholangioscopy in IgG4-related sclerosing cholangitis using texture and color enhancement imaging and red dichromatic imaging. Endoscopy.

[9] Lau W.Y., Fan S.T., Yip W.C., Poon G.P., Wong K.K. (1988). Optimal irrigation pressures in operative choledochoscopy. Aust. N. Z. J. Surg..

[10] Thosani N., Zubarik R.S., Kochar R., Kothari S., Sardana N., Nguyen T., Banerjee S. (2016). Prospective evaluation of bacteremia rates and infectious complications among patients undergoing single-operator choledochoscopy during ERCP. Endoscopy.

[11] Sethi A., Chen Y.K., Austin G.L., Brown W.R., Brauer B.C., Fukami N.N., Khan A.H., Shah R.J. (2011). ERCP with cholangiopancreatoscopy may be associated with higher rates of complications than ERCP alone: A single-center experience. Gastrointest. Endosc..

[12] Mandai K., Uno K., Yasuda K. (2020). Gastrointestinal: Plastic stent-induced polyp-like lesion in the bile duct. J. Gastroenterol. Hepatol..

[13] Caillol F., Bories E., Poizat F., Pesenti C., Esterni B., Monges G., Giovannini M. (2013). Endomicroscopy in bile duct: Inflammation interferes with pCLE applied in the bile duct: A prospective study of 54 patients. United Eur. Gastroenterol. J..

[14] De Vries A.B., van der Heide F., Ter Steege R.W.F., Koornstra J.J., Buddingh K.T., Gouw A.S.H., Weersma R.K. (2020). Limited diagnostic accuracy and clinical impact of single-operator peroral cholangioscopy for indeterminate biliary strictures. Endoscopy.

[15] Yokoe M., Hata J., Takada T., Strasberg S.M., Asbun H.J., Wakabayashi G., Kozaka K., Endo I., Deziel D.J., Miura F. (2018). Tokyo Guidelines 2018: Diagnostic criteria and severity grading of acute cholecystitis (with videos). J. Hepatobiliary Pancreat. Sci..

[16] Liao W.C., Angsuwatcharakon P., Isayama H., Dhir V., Devereaux B., Khor C.J., Ponnudurai R., Lakhtakia S., Lee D.K., Ratanachu-Ek T. (2017). International consensus recommendations for difficult biliary access. Gastrointest. Endosc..

[17] Cotton P.B., Lehman G., Vennes J., Geenen J.E., Russell R.C., Meyers W.C., Liguory C., Nickl N. (1991). Endoscopic sphincterotomy complications and their management: An attempt at consensus. Gastrointest. Endosc..

[18] Kanda Y. (2015). Statistical analysis using freely-available “EZR (Easy R)” software. Rinsho. Ketsueki..

[19] Chen Y.K., Parsi M.A., Binmoeller K.F., Hawes R.H., Pleskow D.K., Slivka A., Haluszka O., Petersen B.T., Sherman S., Deviere J. (2011). Single-operator cholangioscopy in patients requiring evaluation of bile duct disease or therapy of biliary stones (with videos). Gastrointest. Endosc..

[20] Murabayashi T., Ogawa T., Koshita S., Kanno Y., Kusunose H., Sakai T., Masu K., Yonamine K., Miyamoto K., Kozakai F. (2020). Peroral Cholangioscopy-guided Electrohydraulic Lithotripsy with a SpyGlass DS Versus a Conventional Digital Cholangioscope for Difficult Bile Duct Stones. Intern. Med..

[21] Wong J.C., Tang R.S., Teoh A.Y., Sung J.J., Lau J.Y. (2017). Efficacy and safety of novel digital single-operator peroral cholangioscopy-guided laser lithotripsy for complicated biliary stones. Endosc. Int. Open.

[22] Bernica J., Elhanafi S., Kalakota N., Jia Y., Dodoo C., Dwivedi A., Sealock R.J., Patel K., Raijman I., Zuckerman M.J. (2018). Cholangioscopy Is Safe and Feasible in Elderly Patients. Clin. Gastroenterol. Hepatol..

[23] Hammerle C.W., Haider S., Chung M., Pandey A., Smith I., Kahaleh M., Sauer B.G. (2012). Endoscopic retrograde cholangiopancreatography complications in the era of cholangioscopy: Is there an increased risk?. Dig. Liver. Dis..

[24] Kurihara T., Yasuda I., Isayama H., Tsuyuguchi T., Yamaguchi T., Kawabe K., Okabe Y., Hanada K., Hayashi T., Ohtsuka T. (2016). Diagnostic and therapeutic single-operator cholangiopancreatoscopy in biliopancreatic diseases: Prospective multicenter study in Japan. World J. Gastroenterol..

[25] Ghersi S., Fuccio L., Bassi M., Fabbri C., Cennamo V. (2015). Current status of peroral cholangioscopy in biliary tract diseases. World J. Gastrointest. Endosc..

[26] Woo Y.S., Lee J.K., Oh S.H., Kim M.J., Jung J.G., Lee K.H., Lee K.T. (2014). Role of SpyGlass peroral cholangioscopy in the evaluation of indeterminate biliary lesions. Dig. Dis. Sci..

[27] Dumonceau J.M., Kapral C., Aabakken L., Papanikolaou I.S., Tringali A., Vanbiervliet G., Beyna T., Dinis-Ribeiro M., Hritz I., Mariani A. (2020). ERCP-related adverse events: European Society of Gastrointestinal Endoscopy (ESGE) Guideline. Endoscopy.

[28] Angsuwatcharakon P., Kulpatcharapong S., Moon J.H., Ramchandani M., Lau J., Isayama H., Seo D.W., Maydeo A., Wang H.P., Nakai Y. (2022). Consensus guidelines on the role of cholangioscopy to diagnose indeterminate biliary stricture. HPB.

[29] Navaneethan U., Lourdusamy D., Gutierrez N.G., Zhu X., Vargo J.J., Parsi M.A. (2017). New approach to decrease post-ERCP adverse events in patients with primary sclerosing cholangitis. Endosc. Int. Open.

[30] Navaneethan U., Lourdusamy V., Jegadeesan R., Sanaka M.R., Vargo J.J., Parsi M.A. (2015). Su1626 Bile Aspiration During ERCP Is Associated with Lower Risk of Post-ERCP Cholangitis: A Single Center Prospective Study. Gastrointest. Endosc..

[31] Facciorusso A., Ramai D., Gkolfakis P., Khan S.R., Papanikolaou I.S., Triantafyllou K., Tringali A., Chandan S., Mohan B.P., Adler D.G. (2022). Comparative efficacy of different methods for difficult biliary cannulation in ERCP: Systematic review and network meta-analysis. Gastrointest. Endosc..

[32] Navaneethan U., Hasan M.K., Kommaraju K., Zhu X., Hebert-Magee S., Hawes R.H., Vargo J.J., Varadarajulu S., Parsi M.A. (2016). Digital, single-operator cholangiopancreatoscopy in the diagnosis and management of pancreatobiliary disorders: A multicenter clinical experience (with video). Gastrointest. Endosc..

[33] Onoyama T., Takeda Y., Kawata S., Kurumi H., Koda H., Yamashita T., Hamamoto W., Sakamoto Y., Matsumoto K., Isomoto H. (2020). Adequate tissue acquisition rate of peroral cholangioscopy-guided forceps biopsy. Ann. Transl. Med..

